# The "enemies within": regions of the genome that are inherently difficult to replicate

**DOI:** 10.12688/f1000research.11024.1

**Published:** 2017-05-12

**Authors:** Rahul Bhowmick, Ian D Hickson

**Affiliations:** 1 Center for Chromosome Stability, Department of Cellular and Molecular Medicine, Panum Institute, University of Copenhagen, Blegdamsvej 3B, 2200 Copenhagen N, Denmark

**Keywords:** DNA replication, genome, DNA repair machinery, chromosome fragility, replication stress

## Abstract

An unusual feature of many eukaryotic genomes is the presence of regions that appear intrinsically difficult to copy during the process of DNA replication. Curiously, the location of these difficult-to-replicate regions is often conserved between species, implying a valuable role in some aspect of genome organization or maintenance. The most prominent class of these regions in mammalian cells is defined as chromosome fragile sites, which acquired their name because of a propensity to form visible gaps/breaks on otherwise-condensed chromosomes in mitosis. This fragility is particularly apparent following perturbation of DNA replication—a phenomenon often referred to as “replication stress”. Here, we review recent data on the molecular basis for chromosome fragility and the role of fragile sites in the etiology of cancer. In particular, we highlight how studies on fragile sites have provided unexpected insights into how the DNA repair machinery assists in the completion of DNA replication.

## Introduction

The duplication of the genome during the process of DNA replication is fundamental for the viability of all cells. This process initiates from DNA replication origins and proceeds along the parental DNA template in a bidirectional manner until convergence with another replication fork occurs (“DNA replication termination”) ^1^. DNA replication generally occurs with remarkable accuracy. For example, it is estimated that only one error occurs for every 100 million base pairs of replicated DNA in human cells, a degree of fidelity that is achieved by a combination of accurate copying of the template coupled with efficient error correction mechanisms ^2^. Nevertheless, any event that interferes with the DNA replication process has the potential to generate errors that can be inherited by future cell generations. The perturbation of DNA replication in this way is broadly termed “replication stress” ^3^. Although there are several definitions of replication stress, we refer to it as a perturbation of replication fork progression leading to fork stalling and the need for fork protection/repair processes to be employed. An array of highly coordinated DNA repair mechanisms have evolved to ensure that the completion of DNA replication occurs in a timely and faithful manner in order to maintain genomic integrity in the face of replication stress ^4, 5^. Defects in these protective functions can lead to genomic instability and, in multicellular organisms, to cancer predisposition ^6^.

DNA replication stress is implicated as a major driver of tumorigenesis. This is because one of the key features of tumorigenesis is the activation or overexpression of proto-oncogenes, which drives cell proliferation by interfering with the regulatory pathways of cell cycle progression ^7^. Uncontrolled cell proliferation interferes with replication timing and replication fork progression, manifesting as enhanced DNA replication stress ^8^. This can generate chromosome rearrangements and mis-segregation during cell division ^9^, which in turn can lead to aneuploidy (an abnormal number of chromosomes), which is a common feature of solid tumors ^10^. Constitutive activation of a so-called DNA damage response (DDR) during oncogene-induced replication stress was the first evidence to link DNA replication stress with cancer ^11, 12^. This association was strengthened by the detection of mutations in DDR pathway genes in primary cancers ^13– 15^. Moreover, perturbation of DNA replication using agents that slow replication fork progression by inhibiting DNA polymerases (for example, aphidicolin [APH]) can generate DNA rearrangements, such as micro-deletions, that closely resemble those found in human tumors ^16^. Strikingly, recent cancer genome-sequencing projects have revealed that more than half of the DNA rearrangements in cancers cluster within certain chromosomal loci known as common fragile sites (CFSs) ^17^, including in the *FHIT* gene located within FRA3B and the *WWOX* gene within FRA16D. In this article, we discuss recent advances in our knowledge of replication stress and how it particularly affects CFSs.

## Features of common fragile sites

CFSs are regions of the human genome that are present in all individuals ^18^. They were first described over three decades ago as sites where gaps or constrictions are visible in condensed mitotic chromosomes ^19^. Formation of these gaps/constrictions (usually termed fragile site “expression”) is far more prominent in cells exposed to mild replication stress. CFSs are conserved to a large extent in mice ^20– 23^ and other primates ^24^. Over 200 CFSs have been identified in human lymphocytes; however, it is known that the frequency of expression of any single CFS depends on the nature of the replication stress and the cell type. As a result, most of the known sites are infrequently expressed ^25^. Sequence analysis of CFSs has revealed some conserved features. For example, most commonly expressed CFSs harbor a very long gene that takes more than one cell cycle to transcribe. This led to the hypothesis that an underlying cause of CFS expression is the inevitable collision between the replication fork and the transcription machinery that must occur in each S-phase ^26^. Another feature shared in some cases is the presence of stretches of interrupted AT-dinucleotide repeats that influence DNA helix flexibility ^27, 28^ and have the potential to form stable DNA secondary structures ^29^. It was recently demonstrated that replication forks pause at CFS loci in the absence of proteins involved in their maintenance, likely due to the accumulation of DNA-associated structures such as R-loops ^30^. This is proposed to lead to replication fork arrest and DNA breakage. In cultured cells, CFS expression is generally triggered by the use of low doses of APH to induce mild replication stress ^31, 32^. This treatment also induces sister chromatid exchange and an elevated frequency of translocations and deletions at CFSs ^33^. In the context of cancer, CFSs are hotspots for micro-deletions and are associated with chromosomal translocation breakpoints ^34– 38^. CFSs also act as hotspots for viral DNA integration, which can lead to cancer development ^39– 42^. Despite these observations, it is clear that many of the structural changes observed at CFSs in cancers are present on only one allele, and hence these have been proposed to be passenger, not driver, mutations ^33, 37, 38^. Nevertheless, some mouse models with CFS gene inactivation have shown an increased tumor burden ^34, 35^.

## Common fragile site maintenance pathways

Many proteins involved in the recognition and/or repair of DNA damage triggered by replication stress have been reported to play a role in CFS maintenance. These include the ATR and CHK1 kinases, BRCA1, RAD51, and FANCD2 ^30, 43– 45^. A major challenge for CFS maintenance systems in cells undergoing replication stress is ensuring that the completion of DNA replication occurs before the cell enters mitosis. CFSs appear to replicate in late S-phase, which may render them more susceptible to DNA replication stress ^46^. Indeed, they can remain under-replicated and escape the checkpoint surveillance, even when the cell enters mitosis ^47^. This is potentially dangerous, as it can enhance the formation of chromatin bridges, ultra-fine anaphase DNA bridges (UFBs), and lagging chromosomes during mitosis. Anaphase bridges, in turn, can promote the nondisjunction of sister chromatids and micronucleus formation ^47– 49^. Not surprisingly, therefore, cells have evolved efficient mechanisms to process anaphase bridges and counteract mitotic defects. The mechanism of this resolution is poorly characterized but involves the BLM helicase in association with topoisomerase IIIα, RMI1 and RMI2, and the Plk1-interacting checkpoint helicase (PICH) ^49, 50^.

## Common fragile site replication is completed in mitosis during stress conditions

**Figure 1.**
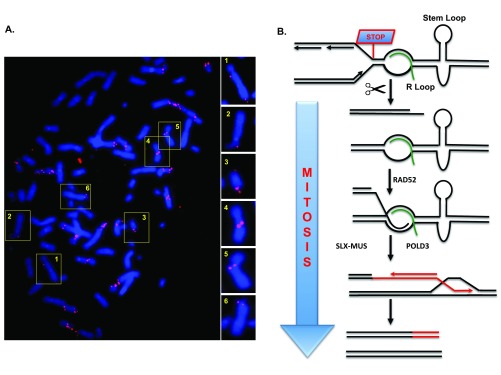
Mitotic DNA synthesis (MiDAS) occurs via a break-induced replication (BIR)-like process. ( **A**) Metaphase spread of U2OS cells treated with low-dose aphidicolin showing mitotic 5-ethynyl-2′-deoxyuridine (EdU) incorporation. Most sites of EdU incorporation exhibit a conservative pattern of DNA synthesis, having EdU incorporation on only one sister chromatid. Selected chromosomes are shown in numbered boxes and are enlarged on the right. ( **B**) Model showing how MiDAS might occur via a BIR-like process. A replication fork stalls at a common fragile site (CFS), perhaps due to the presence of an R-loop or a DNA secondary structure (a stem-loop structure is shown as an example). The fork is then cleaved by an endonuclease, followed by limited end resection of the generated DNA end. This exposes a region of micro-homology that can be annealed with the partially single-stranded template DNA by the RAD52 protein. Processing of the resulting replication intermediate by the activated SLX-MUS complex (SLX4 in complex with MUS81-EME1 and other nucleases) in early mitosis is then associated with POLD3-dependent conservative DNA repair synthesis. This process would account for the high level of copy number variations that arise at CFS loci in cancer cells. For clarity, the replication fork merging with the MiDAS bubble from the right is omitted. If the converging fork were to suffer the same fate as the fork depicted, this could lead to the newly synthesized DNA occurring on both sister chromatids.

Though seemingly counterintuitive, CFS expression is a protective mechanism to promote CFS maintenance ^44^. This can be explained by the fact that CFS expression is not accidental but instead is a programmed event mediated by DNA repair proteins such as the MUS81-EME1 endonuclease ^48, 51^. This finding led us to investigate whether cells might be attempting to rescue failed replication at CFSs in mitosis. Indeed, we demonstrated that replication stress activates nascent DNA synthesis at CFSs in the prophase of mitosis ( Figure 1) ^47^. Interestingly, this synthesis coincides with the sites of the gaps/breaks in the condensed chromosomes. Thus, it would appear that CFS expression denotes sites where DNA synthesis is still ongoing in mitosis. This mitotic DNA synthesis, which we termed MiDAS, has also been detected in other studies using a different kind of replication stress ^52^ and in a different cell type ^53^. Thus, MiDAS is likely to be a universal mechanism used by cells in mitosis to buffer the effects of DNA replication stress.

On the basis of our observations, we propose that MiDAS is not just a continuation of normal semi-conservative DNA replication but rather is a form of DNA repair analogous to break-induced replication (BIR) that has been characterized largely in budding yeast. BIR is generally a conservative form of DNA synthesis, and the nascent DNA is present on only one sister chromatid, leading to the accumulation of changes/mutations in only one allele, as is commonly found at CFSs in cancers ^33, 37, 38, 54^ ( Figure 1). Consistent with this, we have observed characteristic patterns of DNA replication that resemble this in mitotic human cells. MiDAS also requires the RAD52 and POLD3 proteins, the homologs of which (Rad52 and Pol32, respectively) are essential for BIR in yeast ^47, 54– 56^. Indeed, a recent study showed that RAD52 is required more generally for repairing perturbed DNA replication forks in cells exposed to replication stress ^57^. Interestingly, MiDAS in human cells does not require RAD51 ^54^, although most BIR in yeast is Rad51-dependent ^56^.

## Closing remarks

It is curious that MiDAS in human cells is apparently RAD51-independent but that canonical BIR in yeast requires the DNA strand invasion function of this protein. This suggests that MiDAS might represent an atypical form of BIR that occurs in mitosis only at a time when BRCA2 and RAD51 are excluded from the chromatin. One possible explanation for this comes from studies in yeast indicating that RAD51-independent BIR in that organism requires much less homology for DNA copying to be initiated ^58^. As MiDAS occurs during a narrow time window in early mitosis and involves sister chromatids that are already in very close proximity, there might be a selective advantage in using a “quick and dirty” form of repair that serves to prevent the accumulation of fatal mitotic abnormalities at the expense of mutations.

There is still much to be understood about MiDAS; for example, how is the reaction initiated if the replisome remains associated with stalled forks in the CFS loci, and how similar is the mechanism of BIR in human cells to that defined in yeast? Intriguingly, BIR has been proposed to be required for the maintenance of telomeres in cells lacking telomerase, and therefore MiDAS might be mechanistically related to telomere maintenance by the so-called ALT (alternative lengthening of telomeres) pathway ^59^. Moreover, it is possible that the existence of MiDAS could be exploited as a therapeutic approach to kill cancer cells, as MiDAS inhibition is synergistically toxic to cancer cells in combination with inhibitors of ATR kinase ^54^.

Research on CFSs has been ongoing for decades, but these conserved “enemies within the genome” still manage to provide new surprises and insights into the biology of chromosome maintenance. It will be intriguing to define why CFSs have not been eliminated during evolution. Their conservation predicts a positive role in DNA metabolism, but this role remains elusive. Clearly, there is still much to be learned from studying these idiosyncratic regions of our genome.
